# Inorganic Polyphosphate in Mammals: Mechanisms, Maladies, and Moving Forward

**DOI:** 10.3390/biom16010127

**Published:** 2026-01-12

**Authors:** Heala Mendelsohn Aviv, Zhiyun Yang, Zongchao Jia

**Affiliations:** Department of Biomedical and Molecular Sciences, Queen’s University, Kingston, ON K7L 3N6, Canada; 22mhgh@queensu.ca (H.M.A.); zhiyun.yang@queensu.ca (Z.Y.)

**Keywords:** polyphosphate, mitochondria, bioenergetics, coagulation, protein interaction, phase separation

## Abstract

Inorganic polyphosphate is highly conserved, critical, yet poorly understood polymer that regulates diverse cellular functions in mammals. Its importance is well established in coagulation, inflammation, mitochondrial function, and stress responses, though the molecular mechanisms for these effects remain only partly understood. Fundamental questions also persist regarding its physiological concentration, chain-length distributions, and the mechanisms that regulate its behavior in specific cellular compartments. Progress is limited by the absence of a known mammalian polyphosphate-synthesizing enzyme. Despite this, recent studies have broadened the scope of polyphosphate biology, suggesting roles in protein phase separation, ATP-independent chaperone activity, metabolic regulation, and intracellular signaling. Polyphosphate modulates the mitochondrial permeability transition pore through calcium-dependent regulation and activates factor XII in coagulation. Findings have also introduced potential connections between polyphosphate and processes such as neurodegeneration, cancer, and tissue regeneration. Despite this expanding landscape, many biological effects remain difficult to interpret due to incomplete mapping of protein targets and longstanding technical limitations in detecting and quantifying polyP. This review integrates molecular protein-interaction mechanisms with compartment-specific functions and disease physiology, providing a clearer mechanistic framework while identifying key conceptual and methodological gaps and outlining priorities for advancing polyphosphate research in mammalian systems.

## 1. Introduction

**Figure 1:**
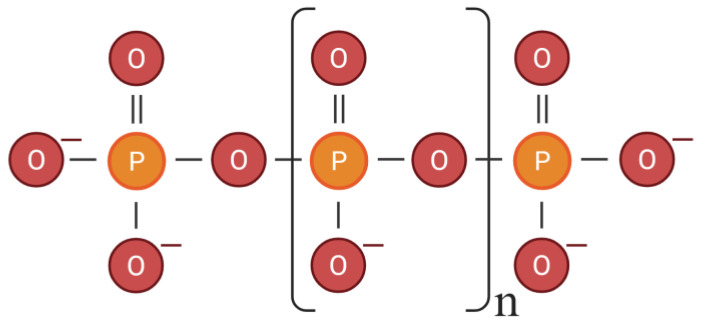
Linear structure of inorganic polyphosphate. The figure was created with BioRender.com.

Inorganic polyphosphate (polyP) (Figure 1) is a linear polymer composed of tens and up to a thousand orthophosphate units joined by high-energy phosphoanhydride bonds [1,2]. Although, polyP is an evolutionarily ancient molecule that is present in all domains of life, early studies contributed to the long-standing assumption that polyP was primarily a microbial metabolite. This perception along with the methodological limitations in its detection led to mammalian polyP research lagging for decades [2,3].

In the past two decades, polyP has emerged as a molecule of significant interest in mammalian biology and medicine. In 2004, Ruiz et al. reported the presence of polyP in human platelets, reporting the first piece of evidence of polyP in human tissues [4]. Shortly after the discovery of polyP in platelets, in 2006, Smith et al. reported on its ability to modulate blood coagulation, linking polyP to this vital mammalian physiological process [5]. These findings proved the presence of polyP in human tissues and reignited interest in polyP’s physiological and pathological function in humans.

Recent research has revealed the presence of polyP in various mammalian cell types and tissues, with different cell types varying in polyP concentration and chain length [6]. Many studies have also found that polyP chains of different lengths play different roles, indicating that the length of the polyP chain influences its function [5,7,8]. Recent research has also implicated polyP in mammalian protein phase separation, cellular signaling, molecular scaffolding, cellular energy metabolism, mitochondrial function, stress responses, inflammation, bone mineralization, and tissue formation [9,10,11,12,13,14,15,16,17]. Other research has also indicated that it potentially plays roles in neurodegeneration and cancer [18,19]. While the many functions of polyP have been increasingly discovered in the past two decades, the mechanisms underlying these diverse functions remain only partially understood.

There are still significant gaps in the scientific community’s understanding of polyP’s synthesis, regulation, and compartment-specific roles. The enzymes involved in both the synthesis and the degradation of polyP are well established in bacteria and *Saccharomyces cerevisiae*, though there is only polyP degrading enzymes known in mammals [20]. This gap in knowledge poses difficulties to the study of polyP, since it makes it difficult to manipulate levels of polyP cells to study its effects.

A mechanistic re-evaluation of polyP is timely. Recent progress has identified many additional mammalian polyP binding proteins and improved the tools used to detect and image polyP. Evidence for its involvement in a range of physiological and disease contexts has also expanded. Earlier reviews have summarized polyP’s known functions and catalogued its roles in specific pathways, but they have not connected these newer mechanistic insights to their physiological and pathological consequences. This review therefore synthesizes recent advances to clarify how protein-level interactions, chain-length-dependent effects, and compartment-specific dynamics converge to shape polyP biology in mammals and highlights the methodological and potential therapeutic directions that follow from current findings.

## 2. Polyphosphate Biology in Mammals

### 2.1. Polyphosphate Synthesis and Degradation

The enzymatic machinery for polyP synthesis in mammalian systems is still poorly defined, especially in comparison with the well-described pathways in prokaryotes and in *S. cerevisiae* [20,21]. In bacteria, polyphosphate kinases (PPKs) catalyse the reversible transfer of γ-phosphate from adenosine triphosphate (ATP) to polyP. In yeast, the vacuolar transporter chaperone subunit 4 (VTC4) catalyzes the transfer of γ-phosphate from ATP to orthophosphate or pyrophosphate [22]. Mammalian orthologs of bacterial PPKs and *S. cerevisiae* VTC4 have not been identified, leading to speculation that alternative enzymes may fulfil the role. In 1963, Lynn et al. found that an excess of electron transport chain substrates in mammalian mitochondria led to much higher levels of polyphosphate, especially when ATP synthesis was inhibited, suggesting a link between polyP synthesis and the enzymes of ATP synthesis [23]. Following this, a study by Baev et al. in 2020 suggested that the mitochondrial ATP synthase (F_0_F_1_-ATP synthase) of mammalian mitochondria produces and hydrolyzes polyP [24]. Baev et al. substantiated these claims through reporting that the inhibition of the F_0_F_1_-ATP synthase with its inhibitor, oligomycin, blocked a rise in polyP levels that adding PO_4_^-^ induced [24]. They also excluded the possibility of the ATP from the ATP synthesis was causing the higher levels of polyP by adding ATP and not seeing an increase in polyP [24]. Ladke et al. further investigated those claims, confirming that orthophosphate (Pi), not ATP, is the source of phosphate for mammalian mitochondrial polyP synthesis [25]. They also provided evidence that polyP levels maintenance relies on both an intact proton gradient across the inner mitochondrial membrane as well as a functional F_0_F_1_-ATP synthase [25]. Kumble and Kornberg’s isotopic-labelling studies in mammalian cell lines also support that intracellular ATP and Pi are unlikely to be the source for the synthesis of polyphosphate in mammals, as polyP synthesis appears to bypass the bulk intracellular pools of these metabolites [6].

Additionally, a study by Reusch et al. from 1998 suggested that the plasma membrane calcium ATPase (PMCA) may function as a polyP kinase in human cells, since it was purified from human erythrocytes [26]. They suggested that PMCA exhibits both ATP synthase activity and ATP/ATP-polyphosphate transferase activity, though this research was, as well, not further substantiated [26]. In principle, it could be rationalized that ATP synthases (including both the F_0_F_1_-ATP synthase and PMCA) could also produce polyP since it would still be forming a phosphoanhydride bond with a substrate and P_i_ using the energy from an electrochemical gradient, only differing in its substrate (polyP_n−1_ vs. ADP).

In both prokaryotes and eukaryotes (including mammals), there are known enzymes capable of degrading polyP. In prokaryotes, polyphosphatases (PPXs) hydrolyse polyP [2]. PPX can function as exopolyphosphatases and endopolyphosphatases, cleaving a P_i_ from the end of a polyP chain and also cleave phosphoanhydride bonds in the middle of the polymers, respectively [2]. *S. cerevisiae* has discrete enzymes for exopolyphospatase and endopolyphosphatases function, unlike prokaryotes. They have exopolyphosphatase PPX1 and endopolyphosphatases PPN1 and DDP1 [22]. Mammalian enzymes with polyphosphatase function have been identified, including those with exopolyphosphatase and endopolyphosphatase function. In mammals, four of the polyphosphatases that have been identified are diphosphoinositol polyphosphate phosphohydrolases (DIPPs), which function as endopolyphosphatases, though other types of mammalian polyphosphatases have also been identified, such as mammalian alkaline phosphatase which acts as an exopolyphosphatase [27,28]. An example of a mammalian exopolyphosphatase is the human metastasis regulator protein, Prune1, which is in the same family as *S. cerevisiae* PPX1 and shares many similarities with bacterial PPX1 [29]. The osteoclast tartrate-resistant acid phosphatase (TRAP) has also shown exopolyphosphatase activity, though it only hydrolyzes shorter-chain polyP and long-chain polyP inhibits this enzyme [30]. Identifying polyP degrading enzymes in mammals is valuable since it supports the discovery of the fuller picture of polyP metabolism and supports further research since it provides a means to manipulate polyP levels.

These findings suggest that mammalian ATP synthases, most notably the mitochondrial F_0_F_1_ complex, could synthesize polyP using Pi as a substrate, although this has not yet been demonstrated with direct biochemical evidence. In contrast, mammalian polyP-degrading enzymes such as DIPPs, Prune1, and TRAP are well characterized. There is a striking imbalance since the mammalian machinery for polyP degradation is clearly defined, yet the origin of mammalian polyP remains uncertain. Elucidating the mammalian source of polyP will be crucial for developing reliable tools to modulate its levels in vivo and for filling one of the major gaps in our understanding of mammalian polyP biology.

### 2.2. Localization and Compartment-Specific Functions of Polyphosphate

PolyP has been detected in various subcellular compartments, at varying concentrations, including mitochondria, plasma membranes, cytoplasm, and secretory granules [4,6]. In mitochondria, polyP has been found to have a role in calcium buffering and has also been found to regulate the mitochondrial permeability transition pore [13,31,32]. Nuclear polyP has been implicated in gene expression by playing a role in chromatin remodelling and transcriptional regulation under normal and stress conditions, though specific molecular targets remain elusive [33,34,35]. PolyP-rich granules are prominent in granule-containing cells, especially in mast cells where polyP is released upon activation of the cells and can contribute to bradykinin-mediated extracellular signaling and procoagulant activity [4,36,37]. In addition to polyP’s roles varying in different subcellular compartments, its content has also been found to vary widely between compartments having been found, in rat hepatocytes, to range from concentrations of 89 ± 7 µM in the nucleus to its lowest concentration in microsomes of 4 ± 0.5 µM [6]. PolyP’s localization patterns and varying concentrations suggest that its function is highly context dependent.

PolyP functions vary across cellular compartments and tissues, though the details of these context-specific roles remain poorly understood [12,38,39]. For example, in the nucleus, polyP has been found in association with phosphorylated nonhistone nuclear proteins and is also known to interact with specific nuclear proteins such as BRD4 and other proteins identified in Table 1, though its precise purpose and nuclear-specific role remain unclear [40,41]. Additionally, the dynamics of polyP trafficking and factors governing its localization are generally unknown, limiting insight into its compartmental roles. Evidence that polyP regulates target proteins under stress suggests that binding may influence its transport, but this possibility has yet to be explored [42]. Overall, understanding polyP’s compartment- and tissue-specific localization is essential as it provides the foundation for the diverse cellular functions and regulatory roles it performs.

### 2.3. Polyphosphate-Protein Interactions: Biochemical and Structural Mechanisms

While previous reviews have addressed polyP localization and function, they often stop short of examining the molecular mechanisms by which polyP exerts its effects in mammalian cells. Here, we intend to focus on emerging evidence of its direct protein interactions, regulatory roles, and energetic functions. PolyP was initially thought to interact covalently with proteins via polyacidic serine- and lysine-rich (PASK) motifs, due to the interaction resisting extreme denaturing conditions, such as 1% SDS or 8M urea [43]. However, recent research by Neville et al. demonstrated that polyP-protein interactions are non-covalent, finding that polyP–protein interactions are ionic strength and pH sensitive [44]. They also found that polyP–protein interactions can occur via repeats of specific amino acids, such as lysine and histidine even in the absence of defined domains such as PASK [9,44]. In addition, proteins with highly positively charged regions can bind polyP, such as proteins with conserved histidine α-helical domains (CHAD) [45]. Though CHADs are not present in mammals, they exemplify electrostatic interactions’ utility in polyP binding that is relevant to mammalian proteins with analogous positively charged regions. These mechanistic insights provide a framework for understanding the diverse set of mammalian proteins now recognized to interact with polyP.

**Table 1:** Experimentally verified mammalian polyP-interacting proteins.

Gene Name	UniProt ID	Type of Interaction	Activity	Citation
*GTF2* *I*	P78347	PASK	Transcription Factor	[46]
*EYA1*	Q99502	PASK	Protein phosphatase and transcriptional coactivator	[46]
*MAFA*	Q8NHW3	His-rich	Transcription Factor	[9]
*MAFB*	Q9Y5Q3	His-rich	Transcription Factor	[9]
*YY1*	P25490	His-rich	Transcription Factor	[9]
*DLX2*	Q07687	His-rich	Transcriptional Activator	[9]
*ZIC3*	O60481	His-rich	Transcriptional Activator	[9]
*GATA6*	Q92908	His-rich	Transcriptional Activator	[9]
*NR4A3*	Q92570	His-rich	Transcriptional Activator	[9]
*POU4F2*	Q12837	His-rich	Transcription Factor	[9]
*OTX1*	P32242	His-rich	Transcription Factor	[9]
*MECP2*	P51608	His-rich	Binds methylated DNA	[9]
*BRD3*	Q15059	Lys-rich	Remodels chromatin structure, regulates transcription	[41]
*NKAP*	Q8N5F7	Lys-rich	Transcriptional repressor	[41]
*CIR1*	Q86X95	Lys-rich	Transcriptional regulator, corepressor with RBPJ	[41]
*TAF3*	Q5VWG9	Lys-rich	Initiates transcription via RNA polymerase II	[41]
*NFRKB*	Q6P4R8-1	Lys-rich	Nuclear factor related to kappa-B-binding protein	[41]
*BBX*	Q8WY36	Lys-rich	Transcription factor	[41]
*ILF3*	Q12906	Lys-rich	RNA-binding protein, role in transcriptional and post-transcriptional processes	[41]
*NCL*	P19338	Lys-rich	Major nuclear protein of growing eukaryotic cells, known role in chromatin decondensation	[41]
*PSIP1*	O75475	Lys-rich	Transcriptional coactivator	[41]
*BRD4*	O60885	Lys-rich	Chromatin reader protein, transmits epigenetic memory	[41]
*MED1*	Q15648	Lys-rich	Component of the coactivator of most RNA polymerase II-dependent genes	[41]
*GTF2F1*	P35269	Lys-rich	Transcription factor	[41]
*UPF3B*	Q9BZI7	PASK	Non-sense mediated mRNA Decay	[47]
*NUFIP2*	Q7Z417	His-rich	RNA Binding	[9]
*MEPCE*	Q7L2J0	His-rich	RNA Methyltransferase	[9]
*SRRM1*	Q8IYB3	Lys-rich	Involved in splicing and binds DNA	[41]
*RBM25*	P49756	Lys-rich	RNA-binding protein	[41]
*HTATSF1*	O43719	Lys-rich	Small nuclear ribonucleoprotein (SnRNP) complex component, role in splicing	[41]
*REXO4*	Q9GZR2	Lys-rich	RNA exonuclease	[41]
*ZRANB2*	O95218	Lys-rich	Splice factor	[41]
*ARGLU1*	Q9NWB6	Lys-rich	Regulates transcription and modulates alternative splicing	[41]
*ZCCHC17*	Q9NP64	Lys-rich	Pnn-interacting nucleolar protein	[41]
*SRPK2*	P78362	Lys-rich	Protein kinase, regulation of splicing	[41]
*ELL2*	O00472	Lys-rich	RNA polymerase II elongation factor	[41]
*DDX55*	Q8NHQ9	Lys-rich	ATP-dependent RNA helicase	[41]
*NOP56*	O00567	PASK	Ribosomal Activator	[47]
*VGLL3*	A8MV65	His-rich	Regulates RNA Polymerase II	[9]
*EIF5B*	O60841	Lys-rich	Translation initiation	[41]
*EIF2B5*	Q13144	PASK	Translational regulation	[46]
*RPL22*	P35268	Not identified	Component of the large ribosomal subunit	[48]
*SDA1*	Q9NVU7	Lys-rich	Role in 60S pre-ribosomal subunit export	[41]
*DEK*	P35659	PASK	Chromatin Organizing	[47]
*TOP1*	P11387	Lys-rich	DNA topoisomerase	[41]
*RRM1*	P23921	Lys-rich	Catalyzes biosynthesis of deoxyribonucleotides necessary for DNA synthesis	[41]
*H1-10*	Q92522	Not identified	Condensation of nucleosome chains into higher order structures	[48]
*CCNT1*	O60563	His-rich	Regulates CDK9 Kinase	[9]
*NKD2*	Q969F2	His-rich	Antagonist of Wnt signaling	[9]
*DYRK1A*	Q13627	His-rich	Protein Kinase	[9]
*RHOBTB2*	Q9BYZ6	His-rich	Guanosine triphosphatase (GTPase)	[9]
*HMGXB4*	Q9UGU5	Lys-rich	Negatively regulates Wnt/β-catenin signaling	[41]
*CSNK1G3*	Q9Y6M4	Lys-rich	Protein kinase, participates in Wnt signaling	[41]
*KRAS*	P01116-2	Lys-rich	GTPase	[41]
*PRICKLE3*	O43900	His-rich	Planar cell polarity pathway	[9]
*RBBP5*	Q15291	Lys-rich	Differentiation of embryonic stem cells	[41]
*GSN*	P06396	PASK	Actin modulation	[46]
*ADD2*	P35612	Lys-rich	Associated with membrane-cytoskeleton	[41]
*CYLC1*	P35663	Lys-rich	Structural role in spermatogenesis	[41]
*ADD3*	Q9UEY8-2	Lys-rich	Associated with membrane-cytoskeleton, role in actin filament capping	[41]
*WASL*	O00401	Lys-rich	Regulates actin polymerization	[41]
*STMN1*	P16949	Not identified	Destabilizing microtubules	[48]
*MESD*	Q14696	PASK	Chaperone, supports Wnt Signaling Pathway	[47]
*HSP90B1*	P14625	PASK	Chaperone, supports Wnt Signaling pathway	[46]
*PPIB*	P23284	Not identified	Protein chaperone -catalyzes peptidyl prolyl cis trans isomerization	[48]
*HRC*	P23327	His-rich	Calcium Binding	[9]
*MICU2*	Q8IYU8	Not identified	Calcium update protein 2	[25]
*ARSJ*	Q5FYB0	Lys-rich	Hydrolase with calcium cofactor	[41]
*HRG*	P04196	His-rich	Regulator in tumor function, immune system, angiogenesis pathways, vascular endothelial growth factor (VEGF) signaling pathway	[49]
*F12*	P00748	Not Indicated	Initiation of blood coagulation	[49]
*NKTR*	P30414	Lys-rich	NK-tumor recognition	[41]
*GLUD1*	P00367	Not identified	Glutamate dehydrogenase 1	[25]
*ATP5F1A*	P25705	Not identified	Alpha subunit of F_1_ complex of F_0_F_1_ ATP synthase	[25]
*NMT2*	O60551	Lys-rich	Myristoylation of certain proteins	[41]
*AP2S1*	P53680	Not identified	Component of the adaptor protein complex 2—function in protein transport	[48]
*PSMD7*	P51665	Lys-rich	Component of 26S proteasome, degradation of ubiquitinated proteins	[41]
*CBLL2*	Q8N7E2	Lys-rich	E3 Ubiquitin-protein ligase	[41]
*ASPH*	Q12797	Lys-rich	Aspartyl/asparaginyl beta-hydroxylase	[41]
*KIAAO753*	Q2KHM9	Lys-rich	Protein moonraker	[41]
*TTC27*	Q6P3X3	PASK	Unknown	[46]

Beyond individual protein targets, polyP has been shown to act as an inorganic molecular chaperone, influencing protein solubility, stability, and phase behaviour, in vitro [11,50,51]. Specifically, Gray et al. showed, in vitro, that long-chain polyP can prevent protein aggregation under stress by acting as a charge-based, ATP-independent molecular chaperone [11]. While this chaperone-like activity has been validated in bacterial and yeast lysates, there is limited direct evidence in living mammalian cells. In contrast, a recent study found that short-chain polyP actively promotes tau fibrillization and neurotoxicity in mammalian neurons [8]. These contrasting findings highlight the context- and length-dependent effects of polyP on protein behaviour. Beyond tau, other disordered or aggregation-prone proteins may also be affected by polyP, especially under stress conditions, and it is important going forward to identify these targets in mammalian cells. Known chaperone protein targets of polyP include Hsp90B and CypB [46].

In addition to modulating protein aggregation, polyP’s ability to engage multiple positively charged protein regions suggests a role in biomolecular condensation. Neville et al. discovered, in HeLa cells, that polyP interactions with DYRK1A disrupted its ability to localize to nuclear speckles, meaning it was not able to take part in phase-separating behaviour which is necessary for many nuclear functions [9]. Additional in vitro studies indicate that polyP can promote phase-separated assemblies depending on chain length and protein context [51]. This indicates that it could contribute to the dynamics of stress granules, nucleoli, or other condensates in mammalian cells. Clarifying how polyP influences these processes in mammalian cells remains an important direction for future investigation.

The scientific community’s understanding of polyP’s interactions and effects remains largely empty. The identification and characterization of polyP’s protein targets through which it exerts its effects is incomplete, with the molecular mechanisms also remaining unknown and physiological relevance unclear. A great challenge faced in studying mammalian polyP is determining the physiological concentrations and polymer lengths across tissues and compartments, as current reported values vary widely due to methodological inconsistencies. The regulation of polyP synthesis and degradation in response to cellular cues (e.g., stress, metabolic demand) remains poorly understood, with no consensus on key regulatory enzymes either. PolyP is also known to modulate various signaling pathways, though its full scope of influence has yet to be established. It is also known to interact with many different proteins, though specificity and physiological relevance of interactions need further validation. The role polyP plays in altering intracellular ATP levels is also unclear, though it has been shown to act as a phosphate donor for ATP synthesis in vitro. PolyP’s significance as a bioenergetic substrate under physiological conditions needs to be fully validated. Clarifying these unknowns is critical to understanding polyP’s true molecular and physiological roles and its potential as a central regulator of cellular metabolism and signaling.

## 3. Functional Roles of Polyphosphate in Cellular Physiology

### 3.1. Transcriptional and Chromatin Regulation

PolyP regulates gene expression at multiple levels. Early studies linked polyP to phosphorylated nuclear proteins, while more recent work has shown it directly inhibits RNA polymerase I and alters nucleolar transcription [34,40]. PolyP also interferes with transcriptional regulators such as Mediator complex subunit 1 (MED1), coactivator Bromodomain-containing protein 4 (BRD4), and transcriptional regulator Yin Yang 1 (YY1) by modulating their phase behaviour and nuclear localization, leading to suppression of super-enhancer-driven-transcription [52].

PolyP targets such as TAF3, GTF2F1, and NFRKB, which are all involved in RNA Polymerase II-mediated transcription, suggest that polyP could directly influence transcription, initiation, or elongation. Additionally, splicing-related proteins such as SRRM1, ZRANB2, and RBM25 suggest a post-transcriptional role for polyP in RNA processing, potentially through modulation of nuclear phase-separated compartments.

There is also evidence that under specific stress conditions, cells will accumulate polyP in their nucleolus and the polyP will colocalize with RNA polymerase I to where rDNA transcription occurs [53]. This aligns with polyP interaction with nucleolar proteins like nucleolin (NCL) and nucleolar protein 56 (NOP56), known polyP targets, which could facilitate the recruitment or retention of polyP in these compartments.

The localization findings by Xie et al. [53] support the idea that polyP may directly regulate transcription by physically interacting with core transcriptional and chromatin-remodeling machinery, such as BRD3, RBBP5, CIR1, and TOP1. Jimenez- Nuñez et al. findings of polyP modulating RNA polymerase I activity support this premise [34]. PolyP binding to RRM1, a subunit of ribonucleotide reductase, is also noteworthy given its role in DNA replication and links to chemotherapy resistance [54]. All of the polyP targets responsible for gene expression further implicate polyP in the regulation of the process, though the mechanisms require further research.

### 3.2. Bioenergetics and Metabolism

The high energy phosphoanhydride bonds in polyP suggest that its function may extend beyond signaling to include fundamental roles in energy metabolism. Müller et al. demonstrated that polyP can act as a direct donor of high-energy phosphate for the enzymatic regeneration of ADP and ATP, implying that it can participate directly in cellular energy buffering and regeneration processes [55]. Schröder et al. proposed a model where polyP plays a role in systemic energy distribution as an energy source that is circulated by platelets and delivered to peripheral sites such as bone defects or injuries, to support the energy-demanding processes needed [56]. The known role of polyP in ADP/ATP regeneration and the proposed role of polyP in energy distribution both position polyP as a bioenergetic resource with broader physiological implications beyond localized signaling.

### 3.3. Mitochondrial Ion Homeostasis and Stress Responses

PolyP has emerged as an important regulator of mitochondrial stress responses and ion homeostasis in mammalian cells. In mitochondria, polyP plays a role in calcium buffering and modulates the mitochondrial permeability transition pore (mPTP), both of which are central regulators of apoptosis, particularly in cardiac myocytes [31,38,57]. Experimental evidence from patch-clamp experiments, enzymatic depletion of polyP, and electron microscopy has shown that polyP sensitizes mPTP to calcium and reactive oxygen species, which promotes its opening under stress conditions [31,38,58]. PolyP also supports mitochondrial calcium uptake and retention, with its removal disrupting sustained mitochondrial calcium elevation after stimulation of the cell [32,59]. The maintenance of the mitochondrial membrane potential and therefore cell survival under stress are both implicated in polyP’s regulation of both mitochondrial free calcium and of mPTP. Furthermore, it has been shown that targeted enzymatic depletion of polyP by polyphosphatases inhibits calcium-induced cell death and alters mitochondrial metabolism [13]. These findings establish polyP as a key factor in maintaining mitochondrial ion homeostasis, membrane integrity, and stress responses in mammalian cells.

## 4. Polyphosphate in Mammalian Disease

### 4.1. Hemostasis and Thrombosis

PolyP plays a critical role in the blood coagulation pathway, driving fibrin formation via interactions with multiple proteins including those in the intrinsic pathway [5,60]. Activated human platelets secrete polyP, which binds and activates Factor XII (FXII), the zymogen that initiates the contact pathway of coagulation including the generation of enzymes activated Factor XII (FXIIa) and kallikrein [37,61,62]. Müller et al. demonstrated this mechanism in vivo, showing that in FXII-deficient plasma, polyP failed to trigger clotting, highlighting FXII as essential for polyP-mediated clotting [37].

PolyP greatly accelerates Factor XI (FXI) activation by thrombin, a reaction that would otherwise be inefficient in solution [63]. This activity supports the understanding of polyP as a key cofactor in hemostasis, since Factor XI (FXI) plays a significant role in controlling bleeding (hemostasis) [64]. Additionally, polyP accelerates thrombin generation since it enhances the rate of Factor V cleavage by activated Factor XI (FXIa), activated Factor X (FXa) and thrombin, a rate-limiting step in thrombin generation [5,65]. PolyP also disables the anticoagulant function of Tissue Factor Pathway Inhibitor (TFPI) and enhances its inactivation by FXIa [5,66,67]. These findings stem from in vitro assays using human plasma or purified systems and are consistent with coagulation behaviour observed in physiological systems [5,37].

PolyP enhances the activity of the antifibrinolytic agent, thrombin-activatable fibrinolysis inhibitor (TAFI) and is incorporated into fibrin structures, resulting in fibrin networks that are more resistant to fibrinolysis [5,64]. The fibrin remodeling effect was observed under physiological conditions and supports polyP’s role in stabilizing clots in living cells [68].

PolyP is implicated in thrombosis through its coagulant function [58]. For example, Malik et al. showed that polyP-induced thrombosis in mice was FXII-dependent and could be attenuated by histidine-rich glycoprotein, which is an established polyP target, providing direct physiological evidence of polyP’s thrombotic role [49]. Moreover, polyP levels are also elevated in certain disease states, such as multiple myeloma, where malignant plasma cells accumulate polyP potentially linking coagulation activity with disease progression and altered nucleolar transcription [34]. Additionally, polyP has been shown to selectively induce apoptosis in human plasma cells, including myeloma cells, an effect observed in primary patient-derived cells [69]. The roles of polyP and those connections all suggest that it is a modulator of coagulation, immunity, and cell survival.

### 4.2. Inflammation and Endothelial Dysfunction

PolyP has well-established proinflammatory properties that extend beyond its contribution to platelet-mediated coagulation. In endothelial cells, polyP promotes the activation of the nuclear factor kappa-light-chain-enhancer of activated B cells (NF-κB) signaling pathway, leading to increased expression of adhesion molecules intracellular adhesion molecule-1 I (CAM-1), vascular cell adhesion molecule-1 (VCAM-1), and E-selectin. This induces adhesion of THP-1 monocytic cell line (THP-1) to the endothelial cell surface [70]. Bae et al. confirmed these responses occur with polyP similar in length to platelet-derived chains, and demonstrated that such polyP induces endothelial permeability and apoptosis in vitro [70]. Importantly, in vivo experiments involving mice showed that injection of polyP in mice led to vascular leakage and leukocyte recruitment, providing direct physiological evidence of polyP’s inflammatory potential [70]. PolyP’s proinflammatory signaling does not depend on TLR2, TLR4, or RAGE, unlike pathogen-associated molecular patterns [70]. This suggests the presence of a unique, yet unidentified, intracellular signaling route. Despite the physiological significance requiring further experimental validation, these findings suggest that polyP influences both hemostasis and inflammation.

PolyP has also been implicated in cardiovascular and neurological pathophysiology. PolyP is specifically implicated during ischemia–reperfusion injury, something mitochondrial dysfunction and cell death play key roles in. In cardiac myocytes, polyP has well-established roles, as previously discussed, in regulating mitochondrial permeability transition pore and mitochondrial calcium buffering, acting as a modulator of calcium-dependent apoptosis and necrosis during oxidative stress [58,71]. These findings suggest a role for polyP in driving tissue damage in ischemic heart disease, potentially linking it to inflammation-associated thrombosis, which is a role that is supported by Malik et al.’s [49] findings of polyP inducing thrombosis. In a neuroinflammatory context, Arredondo et al. demonstrated that astrocytes, derived from Amyotrophic Lateral Sclerosis (ALS)/Frontotemporal Dementia (FTD) patients, secrete excess polyP, which was found to induce non-cell-autonomous motoneuron death [72]. Notably, when polyP was degraded or neutralized, the toxicity was found to be weakened, implicating polyP itself as a key mediator [72]. Intracellular and secreted polyP are therefore implicated in inflammation by these findings, particularly under disease-associated stress conditions. Overall, these connections demonstrate the physiological harms that polyP may cause in living systems through coagulation and inflammation processes.

### 4.3. PolyP Connection to Neurodegeneration

PolyP is heavily implicated in neurodegenerative disease in mammalian systems through its roles in neuroinflammation, protein aggregation, and amyloid fibril formation. A study performed by Cremers et al. demonstrated that polyP acts as a conserved modulator of amyloidogenic processes, where it accelerates amyloid fibril formation and reduces intermediate toxicity [73]. These researchers also found a significant decline in brain polyP levels of mice with amyloid pathology in comparison to age-matched wild-type controls [73]. This study’s findings have heavy implications for Alzheimer’s disease, supporting the idea that polyP is dynamically regulated during disease progression and can be effectively studied using murine models. Müller et al. explored the neuroprotective effect of polyP mechanistically, using rat primary cortical neuronal cells, and found that the process of polyP protection occurred through the recovery of cellular energy status compromised by β-amyloid-induced ATP depletion (specifically, the Alzheimer peptide amyloid-beta (Aβ) 25–35) [74]. This mechanistic insight is complimented by the work of Lempart et al. who found that polyP modifies fibril formation of disease-related amyloids and prevents the uptake of fibrils into neuronal cells, acting to promote neuroprotection [18]. This work specifically focuses on the amyloid protein, α-synuclein, which plays a key role in Parkinson’s disease, and positions polyP as a potential therapeutic target. PolyP may not only play a role itself in modifying protein aggregation, but it may also interact with proteins that are responsible for assisting in protein folding and preventing aggregation, such as Hsp90, a protein chaperone and also an established polyP target. In contrast to polyP’s neuroprotective role, Barolo et al. revealed that short-chain polyP species induce tau fibrillation and neurotoxicity in human iPSC-derived neurons, revealing the neurotoxic role polyP can play and uncovering its connection to diseases with tau fibrillation such as Alzheimer’s and frontotemporal dementia (FTD) [8]. Together, these studies position polyP as a critical regulator of protein aggregation and subsequent disease, with length- and molecular context-dependent functions.

Beyond protein aggregation, dysregulated polyP has also been linked to other neurodegenerative pathways. The findings by Arrendondo et al. showing that disease-state (ALS/FTD) astrocytes release an excess amount of polyP, causing non-cell-autonomous toxicity to motoneurons, further displays the pathogenic potential of polyP in neurodegenerative contexts [72]. In contrast, Maiolino et al. demonstrated that polyP protects against glutamate-induced excitotoxicity, which is a great contributor to neuronal cell death in many neurodegenerative disorders, such as Alzheimer’s disease, Huntington’s disease, Parkinson’s disease, and ALS [75]. PolyP plays a neuroprotective effect in this way by modulating α-amino-3-hydroxy-5-methyl-4-isoxazolepropionic acid (AMPA) and N-methyl-D-aspartate (NMDA) receptors via P2Y purinergic receptor (P2Y) receptor activation, displaying an alternative mechanism of its neuroprotective function [75]. The role polyP plays in preventing glutamate-induced excitotoxicity is significant due to the glutamate excitotoxicity being a shared pathological pathway of numerous neurodegenerative diseases, displaying the pathophysiological significance of polyP in mammalian neurology.

### 4.4. PolyP Role in Cancer

PolyP has been found to play roles in tumor growth and metastasis through modification of cellular metabolism, signaling pathways, and cellular proliferation in mammalian cancer models. PolyP has been found to activate transient receptor potential melastatin 8 (TRPM8) signaling and therefore promote colorectal cancer growth [76]. Wang et al. found, in vitro and in vivo, that polyP is a potent stimulator of mammalian target of rapamycin (mTOR), a eukaryotic kinase involved in cellular proliferation and heavily implicated in cancer, though shorter polyP does not show the same effect [77]. PolyP depletion by yeast polyphosphatase (PPX1) in MCF-7 mammary cancer cells resulted in reduced insulin and amino acid activation of mTOR activity. Additionally, they found that their overall size and proliferation was affected in serum-free medium (though these were not dramatically affected in serum-containing medium) [77]. These findings indicate that the polyP regulation, implicated by the results of the exopolyphosphatase activity, appears to be a stress response to nutritional deficiency, positioning polyP as a tumor metabolism regulator. Bru et al. found that polyP is essential for survival after DNA damage, highlighting its role as a conserved stress-response factor, which is highly relevant given that tumors face many stresses such as hypoxia, oxidative stress, and upon treatment with chemotherapy drugs such as gemcitabine, DNA damage [35,54]. Boyineni et al. findings support this since they show that polyP is present at high levels in several types of cancer cells of different origins [19]. They also proposed that polyP may be utilized as a source of phosphate energy in cancer. Using brain tumor-initiating cells (BTICs) derived from a mouse brain tumor model, they found that polyP was depleted during starvation and during manipulated ATP depletion [19]. Additionally, they found that enzymatic hydrolysis of polyP impaired cancer cell viability and significantly decreased ATP stores [19]. Another connection between polyP and ATP in mammalian cancer cells was found by Müller et al., that was that the addition of synthetic polyP to human osteogenic sarcoma cells led to ADP and ATP accumulation in the extracellular space [55]. These findings are in agreement with the suggestion by Boyineni et al. [19] of a common role of polyP in tumorigenesis as a phosphate energy source. While some studies suggest polyP promotes tumor growth through metabolic regulation, others point to an anti-metastatic role, specifically in melanoma. Specifically, Han et al. found, in murine melanoma cells, that polyP effectively blocking tumor cell proliferation by inhibiting DNA synthesis and therefore neovascularization, playing an anti-metastatic role in this context [78].

**Figure 2:**
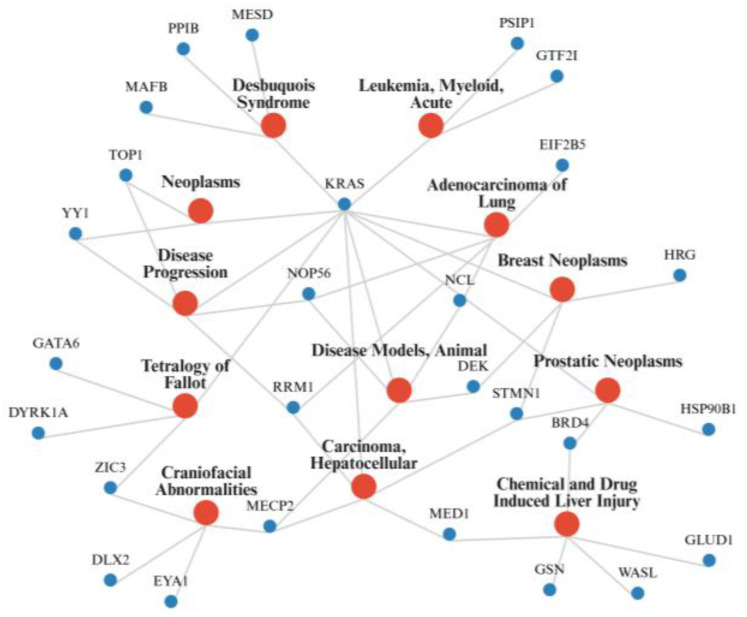
Shared Disease Connections of PolyP Interacting Proteins. A disease-gene network was generated using curated associations from the Comparative Toxicogenomics Database (CTD) and the final figure was created with BioRender.com [79]. Nodes representing genes encoding mammalian proteins with validated polyP binding from Table 1 (blue), and disease terms linked to those genes (red). Edges denote CTD-curated relationships between each polyP-interacting gene and its associated disease.

To contextualize how polyP-interacting proteins align with mammalian disease biology, a network was generated using curated gene-disease associations from the Comparative Toxicogenomics Database (CTD) (Figure 2) [79]. The resulting map reveals prominent clustering across neoplastic disorders, developmental syndromes, and chemically induced liver injury. This suggests that polyP-binding proteins participate in many common disease mechanisms.

### 4.5. PolyP Role in Viral Defense

PolyP plays established roles in many types of diseases that have been explored in this review, though others such as SARS-CoV-2 and human immunodeficiency virus type 1 (HIV-1) have yet to be discussed. Lorenz et al. found that longer-chain polyPs (mean chain lengths of 15 and 34) act in an anti-HIV manner, in vitro, by binding both the host cell surface and the virus, thereby inhibiting absorption of the virus [80]. More recently, polyP at physiological concentrations was found to prevent SARS-CoV-2 infection and replication, in human embryonic kidney and monkey kidney cells [81]. PolyP was found to do this by degrading the host-cell receptor, angiotensin-converting enzyme 2 (ACE2), extracellularly, and the SARS-CoV-2 RNA-dependent RNA polymerase (RdRp) protein, intracellularly, through protease-mediated degradation [81]. Additionally, through its role in modulating the NF-κB inflammatory pathway, longer chain polyP was found to decrease the expression of many of the cytokines that are highly expressed as a part of the inflammatory response seen in patients with COVID-19 [81]. PolyP’s prevention of SARS-CoV-2 infection presents a new role for polyP as a member of the host’s antiviral innate immune defense.

The role polyP plays in coagulation and immune-related disease is well-established, though its involvement in complex pathologies like neurodegenerative diseases, cancer, and chronic inflammation is only beginning to be appreciated and is the focus of ongoing research [15,19,72]. Although the emerging findings are compelling, they are largely descriptive and appear contradictory in some cases. For example, polyP can be neuroprotective or neurotoxic and can promote or inhibit tumor progression depending on the context. These discrepancies can be reconciled by considering four key variables that govern polyP function: chain length, concentration, subcellular localization, and the physiological state of the target tissue [12,38,39]. Importantly, many of these studies require replication, particularly in physiologically relevant models, to validate observed effects and assess their potential as therapeutic targets. The elucidation of the precise mechanisms and physiological contexts of polyP’s actions in these diseases has great potential for revealing fundamental mammalian biology and potential therapeutic strategies.

### 4.6. Therapeutic Relevance

PolyP-based therapeutics are being explored across diverse disease contexts, including regenerative medicine, cancer therapy, and infectious disease. While most approaches remain in the preclinical stage, several lines of evidence point to both clear biological effects of polyP and translational promise.

PolyP shows particular potential in modulating coagulation, promoting tissue regeneration, and supporting bone and cartilage formation [82,83,84]. For example, amorphous calcium polyphosphate nanoparticles have been shown to regulate intracellular ATP levels in osteoblast-like cells, which suggests applications in bone regeneration through contributing not only structural support but also metabolic enhancement for bone growth [85]. The biological outcomes of polyP formulations can vary depending on the associated cationic counterion: Mg^2+^-containing particles tend to favor cartilage repair, whereas calcium or strontium versions enhance bone formation [84,86]. These materials have also been reported to alter the expression of genes at the core of skeletal tissue formation, including osteocalcin, collagen 3A1, collagen 2, SOX9, aggrecan, and alkaline phosphatase [16,86].

PolyP-based systems are also being tested in wound-healing contexts. Both in vitro and in vivo models demonstrated accelerated re-epithelialization and improved cellular energy status following polyP treatment [85,86]. These outcomes likely stem from several intersecting mechanisms including polyP’s ability to act as a phosphate donor, regulate intracellular ATP, and influence signaling pathways that govern differentiation and extracellular-matrix production [85,86].

In the contexts of cancer, polyP can modulate cellular metabolism and influence disease-related pathways. For instance, in preclinical models, long-chain polyP administration reduced lung metastases in murine melanoma, suggesting a direct mechanistic role in tumor progression and angiogenesis [19,78,87]. PolyP has also been studied as a chemotherapy adjuvant, where it enhanced cisplatin sensitivity, pointing to a role in modulating drug responsiveness in cancer therapy [53]. Notably, RRM1, an established polyP target is a kye determinant of chemotherapy drug Gemcitabine sensitivity, further underscoring how polyP-protein interactions may intersect with cancer therapeutics [54].

PolyP is also being explored in the context of infectious diseases and acquired immune deficiency. In one of the earlier studies, linear inorganic polyphosphates of chain lengths about 15,34, and 91 phosphate residues inhibited HIV-1 infection in vitro, preventing viral absorption[80]. PolyP has also been explored as in antiviral strategies, as it has been shown that long-chain polyP impairing viral infection and replication in SARS-CoV-2 models [81].

Other preclinical investigations extend polyP’s therapeutic potential into metabolic and neurodegenerative disease. In diabetes, polyP has been reported to restore the impaired metabolic energy balance in endothelial cells that is caused by high glucose in diabetes, which sometimes can lead to conditions like necrosis [88,89]. In neurodegeneration, it mitigates β-amyloid-induced ATP depletion and suppresses downstream neurotoxic effects, suggesting relevance to conditions such as Alzheimer’s disease [74].

Significant translation barriers remain in the way of extending these promising preclinical findings into a clinical setting. One of the barriers includes that therapeutic activity depends heavily on polyP chain length, yet producing polymers of defined chain length and purity is technically difficult [90,91]. Another barrier is that polyP is rapidly degraded by endogenous phosphatases, creating stability and delivery constraints in vivo [27,29]. PolyP’s pleiotropic effects across coagulation and inflammation highlight the need for targeted delivery to avoid off-target activation [5,70]. Finally, the absence of a known mammalian polyP-synthesizing enzyme limits genetic or enzymatic approaches for modulating endogenous levels [20]. It will be necessary to address these challenges in order to translate polyP’s regenerative, antiviral, and anticancer potential into clinically viable strategies.

**Figure 3:**
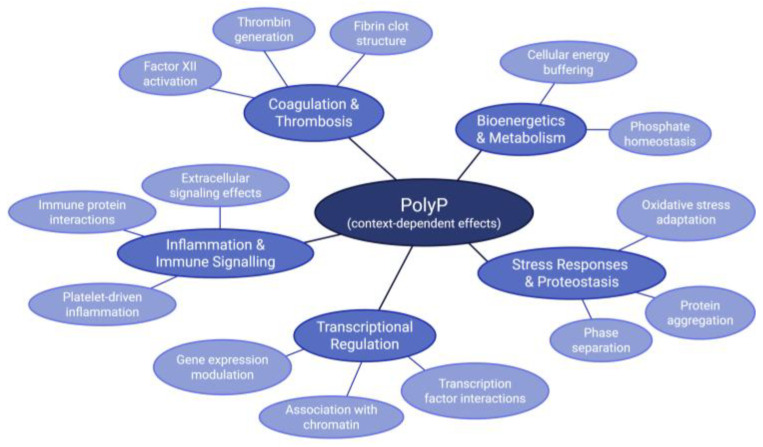
Involvement of PolyP in Selected Disease and Physiological Processes. A schematic overview summarizing disease-related and physiological processes involving polyP that are discussed in this review. PolyP is depicted as a central node linked to associated processes. Secondary nodes represent contexts in which polyP plays a role in mammalian biology, with the tertiary nodes representing a few of the ways polyP is involved in each context. The figure highlights key ways that polyP is involved in the different physiological and disease contexts selected for this figure, rather than depicting all reported mechanisms within each process. The figure was created with BioRender.com.

These findings are all important in contributing to the broad and growing preclinical foundation of mammalian polyP research. Mechanistic elaboration provide insight into how polyP influences energy metabolism, signaling, and matrix biology. This insight provides a basis for its translational promise across coagulation, regeneration, cancer, infection, and beyond. A selection of polyP’s involvements in disease and physiological processes is illustrated (Figure 3) to provide a visual synthesis of the functional contexts discussed in this review.

## 5. Methodological Limitations of Polyphosphate Research

Reported levels of polyP in mammalian cells vary widely across the literature, with differences exceeding what would be expected from normal biological variability. For instance, in human embryonic kidney (HEK293) cells, Kumble et al. reported polyP levels as low as 0.31 nmol Pi/mg protein using enzymatic hydrolysis with *Escherichia coli* PPK and yeast rPPX1 to convert polyP into quantifiable products [6]. In contrast, Borghi et al. used a PPX1-malachite green assay in TREx-293 (HEK293-derived) cells and detected significantly higher levels of endogenous polyP concentrated in the nucleolus [39]. Such variability is unlikely to reflect true differences in cellular content, alone, and instead underscores how assay choice and detection chemistry strongly influence measurement values [92]. These inconsistencies highlight the impact of methodological limitations and the need for the standardized, validated methods of polyP quantification in mammalian systems.

Accurate detection and quantification of polyP in mammalian cells have historically been performed using fluorescent reporters, though this method faces issues of interference and requires further progress. The most common method of polyP assessment is use of 4′,6-diamidino-2-phenylindole (DAPI)-based fluorescence assays where total polyP content can be measured by fluorescence, in living cells [25,90]. This method is not entirely reliable as it suffers from poor specificity due to cross-reactivity with nucleic acids and other polyanions, leading to unreliable and inconsistent results across studies [93]. To overcome the limitations of low specificity, fluorescent polyP reporters JC-D7 and JC-D8 have been proposed since their specificity for polyP is higher, suggesting that there would be likely less interfering signals [94]. These alternative reporters face issues of low binding affinity to polyP, making them also somewhat unreliable as well [94]. Furthermore, the affinity of these probes in particular varies with chain length, making results dependent on the underlying polymer distribution [94]. Despite their drawbacks, JC-D7 and JC-D8 have enabled real-time visualization of endogenous polyP dynamics in mammalian cells with greater selectivity than DAPI, demonstrating real incremental progress. Limitations to microscopy-based polyP detection generally includes that cells often require fixation and multiple washing steps, which can lead to artifactual decreases in measurable polyP content, limiting the accuracy of imaging-based quantification. Overall, fluorescent reporters are valuable tools since they can be used to assess polyP in mammalian cells, non-destructively, and provide information about location and quantity of polyP, though evidently there needs to be more progress on their precision and accuracy before they can be used in isolation of other methods.

Enzymatic approaches have been central to polyP quantification for a long time, and many early mammalian studies adapted methods first used in microbes. For example, Kumble et al. used recombinant *E. coli* PPK and yeast rPPX1 to hydrolyze polyP in HEK293 cell extracts, releasing phosphate for subsequent quantification. A way this approach has been altered is by using yeast exopolyphosphatase ScPpx1p, with pyrophosphatase scIpp1p being used since it can resolve polyP down to very short chain lengths [91,95]. Additionally, those two enzymes are useful since they are less affected by interference from other phosphorus-containing metabolites [91,95]. However, these assays’ use is limited in mammalian systems since the assays are destructive, depend on enzyme specificity, and require reagents that are not widely available.

There are more advanced approaches for in situ polyP imaging beginning to emerge, including genetically encoded biosensors. One promising direction is fluorescence resonance energy transfer (FRET)-based phosphate sensors, which may eventually be adapted for polyP detection [96]. A more direct demonstration of genetically encoded strategies was pioneered by Samper-Martín et al. [14], who expressed DNA constructs coding for the polyP-binding domain (PPBD) of *E. coli* Ppx1 in mammalian cells. This enabled the biosensor to report on the presence, localization, and dynamics of polyP with much greater specificity than fluorescent reporters, representing a significant advance in polyP visualization in living cells [14,97]. This method has been used successfully in mammalian cells, including HEK293T, to quantify nuclear polyP with high specificity [14,97,98]. This method has its limitations as well, providing only a partial picture, as it is restricted to the nucleus and cannot measure polyP in other cellular compartments [97].

Advanced analytical methods offer the potential for more precise quantification and chain length resolution, though their application to mammalian cells has remained limited. A non-destructive analytical that is used to quantify polyP in living cells is nuclear magnetic resonance (NMR) spectroscopy, which has been used successfully to provide precise quantification in intact yeast and fungal cells, though has not yet been applied to mammalian cells. [99,100,101]. Other methods such as end-point titration, polyacrylamide gel electrophoresis (PAGE), and high-throughput liquid chromatography (HPLC) have been successfully used on purified mammalian cell lysates, though they are limiting since they cannot assess polyP in living cells [91,102,103].

The first primary challenge in studying mammalian polyP is the absence of clear homologs of the polyP polymerases that are well characterized in microbes, such as bacterial PPK and the yeast VTC4 subunit [1,20]. As a result, most of the mechanistic framework for polyP biology is derived from yeast and bacterial systems. In mammalian systems, however, the lack of identified polymerases makes it technically difficult to manipulate intracellular polyP levels genetically. To overcome this limitation, researchers often rely on the heterologous expression of microbial enzymes [13]. This approach is flawed, as heterologous expression of microbial enzymes can lead to artifacts such as cellular toxicity, imbalanced or excessive polyP accumulation, and localization to compartments where polyp would not normally be synthesized in mammalian cells [13,14,27,39].

A second challenge in studying mammalian polyP is the variability in polyP content and distribution across mammalian cells and tissues. There is currently no clear baseline of what is physiologically relevant, making it difficult to interpret differences between cell types or to generalize results from cultured cells to whole organisms [6,34,39,92]. Lack of easily available standardized polyP reference material, such as polyP of defined chain length and purity, also acts as a barrier as well, limiting consistency and comparability across studies [90,91].

The major polyP detection and quantification strategies used in the literature are summarized to help clarify how methodological variability contributes to inconsistent polyP measurement in mammalian systems (Table 2). These approaches differ widely in sample requirements, specificity, and suitability for live-cell analysis. Each method carries limitations that directly impact reported polyP levels and therefore interpretation of biological results.

## 6. Discussion and Future Directions

The priority for advancing polyP detection technologies is the development of specific, sensitive detection methods. Genetically encoded biosensors reflect a particularly promising direction. They offer the potential for real-time, compartment-specific quantification in living cells and organisms. Among these biosensors, FRET-based conformational biosensors show a potential implementation of these tools, since the biosensors could be engineered to detect polyP. Similar to polyP detection, Banerjee et al. developed a genetically encoded FRET biosensor to visualize inorganic phosphate dynamics in Caenorhabditis elegans [96]. The successful implementation of these tools demonstrates a potential for extending this conceptual framework to polyP and opening the door to in vivo monitoring of this polymer.

Single-fluorophore sensor with circularly permuted fluorescent protein (cpFP) is another method that could be adapted to detect polyP specifically and sensitively in living cells or organisms. This detection method has been used successfully with calcium where it provided clear, sensitive detection of dynamic changes in intracellular levels [104]. Another example of this method being used is with cpGFP in *E. coli* to detect glycolate production, where it enabled enhanced quantification of metabolite levels [105]. This method has also been successful in detecting ATP, which shares the property of having phosphoanhydride bonds with polyP, though this study did struggle with the specificity of the binder since ADP also bound to it and caused false positive detection [106]. Together, these examples highlight the success of cpFP-based sensors and their utility for dynamic, sensitive detection of metabolites and signaling molecules in living systems, a property that would be particularly valuable for polyP.

Another promising approach is using fluorophores that exploit excited-state intramolecular proton transfer (ESIPT). Santra et al. demonstrated its utility, in vitro, as a sensitive probe for inorganic and biological phosphates [107]. More recently, Roy et al. reported ESIPT-based probes for short-chain polyP, where the mechanism of fluorescence provided a distinct “turn-on” signal with strong selectivity for polyP, compared to ATP, ADP, or inorganic phosphate [108]. This design was also able to be used to monitor polyP degradation by exopolyphosphatases, in vitro [108]. These studies establish ESIPT fluorophores as a selective and sensitive chemical strategy for detecting short-chain polyP in controlled assays, without the need for purification. While they have not yet been applied across longer chain lengths of polyP or in living systems, ESIPT probes illustrate a complimentary chemical platform that, if expanded to in vivo contexts, could broaden the toolkit for polyP detection alongside biosensor-based approaches.

Transcriptional reporter systems are also detection systems that could be modified to detect polyP. This technology was used by Li et al. for sensitive detection of cellular c-di-GMP levels in *E. coli* [109]. These systems use transcription factors that activate a reporter upon ligand binding, effectively amplifying the detection signal. For polyP, which is known to target multiple transcription factors (such as GTF2I, MAFA, MAFB, and YY1), this could provide a valuable way to convert polyP binding into strong and quantifiable outputs (Table 1). By coupling polyP-TF interactions to reporter expression, such systems could present the opportunity for dynamic and sensitive monitoring of polyP levels and localization in living cells and organisms.

Robust mammalian disease models, such as murine models, for neurodegeneration, cancer, and inflammation, are essential for linking polyP dynamics to disease. For example, Cremers et al. [73] compared brain polyP levels in human amyloid precursor protein (hAPP) J20 transgenic mice and age-matched controls, finding a significant decline in polyP in the amyloid-bearing mice. This drastic decrease connects polyP regulation to Alzheimer’s disease progression. Many mechanistic studies, such as those by Müller et al., have demonstrated how polyP can modulate cancer cell viability in vitro or ex vivo [87]. This indicates a need to utilise robust mammalian disease models to extend these finds, in order to test the therapeutic effects in vivo.

The future of polyP research hinges on collaboration across chemistry, cell biology, and medicine. In order to uncover polyP’s full potential, it will help to integrate chemical biology, advanced imaging, and system approaches. The standardization of methods necessitates a collaborative effort and will then enhance reproducibility and accelerate progress.

PolyP is a versatile, ubiquitous, evolutionarily conserved polymer becoming increasingly recognized and studied in mammalian biology and disease. There have been recent advances in the field, although gaps in understanding of synthesis, regulation, mechanisms, and disease connections persist. Additionally, progress is hindered by methodological limitations, though emerging technologies offer hope. Addressing challenges in polyP research will require the use of new tools, models, and interdisciplinary efforts and will make it possible to make progress in understanding polyP’s multifaceted roles and potential applications. A deeper understanding of polyP will act to enable therapeutic innovation and also, importantly, illuminate fundamental aspects of mammalian cell biology.

## 7. Conclusions

PolyP is an evolutionarily conserved polymer that is implicated in multiple aspects of mammalian physiology and disease. Evidence from biochemical, cellular, and disease-focused studies indicates that polyP exerts many of its observed effects through direct and indirect interactions with proteins involved in processes such as metabolism, stress responses, coagulation, and transcriptional regulation. While its biological importance is increasingly evident, the molecular mechanisms underlying polyP’s function remain incompletely defined. This review synthesizes current knowledge of polyP synthesis, degradation, localization, and protein interactions, and highlights how these interactions may contribute to physiological regulation and pathology. Additionally, the review highlights the methodological and mechanistic gaps that must be addressed to advance the field.

## Figures and Tables

**Table 2:** Capabilities and limitations of methods for measuring polyphosphate.

Method	Description	Sample Requirement	Specificity	In Vivo Compatible	Major Limitations	Citation
DAPI Fluorescence	Detect polyP by DAPI emission shift	Live or fixed cells	Low	Yes	Cross-reacts with nucleic acids and chain length bias	[90,93]
JC-D7/JC-D8 Fluorescent Probes	Fluorescent probes with higher selectivity for polyP	Live or fixed cells	Moderate	Potentially	Low affinity for polyP, chain-length dependent	[94]
Enzymatic Hydrolysis	Recombinant microbial polyphosphatases degrade polyP into Pi for quantification	Cell lysates	High	No	Destructive and depends on microbial enzymes	[6,91]
PPX-Malachite Green Assay	Yeast PPX1 degrades polyP and released Pi is measured by malachite green colorimetry	Cell lysates	High	No	No compartment resolution	[39]
Genetically Encoded PPBD Sensors	PPBD expressed in cells binds endogenous polyP for detection	Live cells (nucleus only)	High	Yes	Restricted to nuclear polyP and compartment bias	[14,97]
NMR Spectroscopy (^31^P-NMR)	Direct non-destructive polyP detection	Live microbial cells	Very high	Potentially	Not yet applied to mammalian polyP and sensitivity limitations	[99,100]
PAGE/HPLC	Separates polyP by size/chain length	Purified cell lysates	High	No	Requires extraction so cannot measure in living cells	[91,103]

## Data Availability

No new data were created or analyzed in this study. Data sharing is not applicable to this article.

## Abbreviations

The following abbreviations are used in this manuscript:

ACE2	Angiotensin-converting enzyme 2
AD	Alzheimer’s disease
ALS	Amyotrophic lateral sclerosis
AMPA	α-amino-3-hydroxy-5-methyl-4-isoxazolepropionic acid receptor
CHAD	Conserved histidine α-helical domain
DAPI	4′,6-diamino-2-phenylindole
DIPP	Diphosphoinositol polyphosphate phosphohydrolase
FXI	Factor XI
FXIa	Activated Factor XI
FXII	Factor XII
FXIIa	Activated Factor XII
FRET	Förster resonance energy transfer
HIV-1	Human immunodeficiency virus type 1
HPLC	High-performance liquid chromatography
mPTP	Mitochondrial permeability transition pore
mTOR	Mammalian target of rapamycin
NMDA	N-methyl-D-aspartate receptor
P2Y	P2Y purinergic receptor
PASK	Polyacidic serine- and lysine-rich motif
Pi	Orthophosphate
PMCA	Plasma membrane calcium ATPase
polyP	Inorganic polyphosphate
PPX	Exopolyphosphatase
RdRp	RNA-dependent RNA polymerase
ROS	Reactive oxygen species
SARS-CoV-2	Severe acute respiratory syndrome coronavirus 2
TFPI	Tissue factor pathway inhibitor
VE GF	Vascular endothelial growth factor
